# Hyper memory, synaesthesia, savants Luria and Borges revisited

**DOI:** 10.1590/1980-57642018dn12-020001

**Published:** 2018

**Authors:** Luis Fornazzari, Melissa Leggieri, Tom A. Schweizer, Raul L. Arizaga, Ricardo F. Allegri, Corinne E. Fischer

**Affiliations:** 1 MD, FRCPC. Department of Medicine, Behavioural Neurology Division, University of Toronto, Toronto, ON, Canada, M5S 1A8. St Michael’s Hospital Memory Disorders Clinic, St. Michael’s Hospital, Toronto, ON, Canada, M5S 1A8. Faculty of Medicine, Department of Psychiatry, University of Toronto, Canada, M5S 1A8.; St. Michael’s Hospital, St Michael’s Hospital Memory Disorders Clinic, Toronto, ON, Canada; University of Toronto, Faculty of Medicine, Department of Psychiatry, Canada; 2 BSc. Keenan Research Centre for Biomedical Research, Li Ka Shing Knowledge Institute, St. Michael’s Hospital, 209 Victoria Street, Toronto, ON, Canada M5B 1T8. Institute of Medical Sciences, University of Toronto, Toronto, ON, Canada, M5S 1A8.; University of Toronto, Institute of Medical Sciences, Toronto, ON, Canada; 3 PhD. Department of Surgery, Division of Neurosurgery, University of Toronto, Toronto, ON, Canada, M5S 1A8. Keenan Research Centre for Biomedical Research, Li Ka Shing Knowledge Institute, St. Michael’s Hospital, 209 Victoria Street, Toronto, ON, Canada M5B 1T8.; St. Michael’s Hospital, Li Ka Shing Knowledge Institute, Keenan Research Centre for Biomedical Research, Toronto, ON, Canada; 4 MD. Faculty of Medicine, University of Buenos Aires, Viamonte 430, 1053 Buenos Aires, Argentina.; 5 MD. Centro de Memoria y Envejecimiento. Ins Investigaciones Neurol “Raul Carrea” (FLENI) Buenos Aires, Argentina. Neurosciencias. Universidad de la Costa (CUC). Colombia.; Universidad de la Costa, Neurosciencias, Colombia; 6 MD, FRCPC. St Michael’s Hospital Memory Disorders Clinic, St. Michael’s Hospital, Toronto, ON, Canada, M5S 1A8. Faculty of Medicine, Department of Psychiatry, University of Toronto, Canada, M5S 1A8. Keenan Research Centre for Biomedical Research, Li Ka Shing Knowledge Institute, St. Michael’s Hospital, 209 Victoria Street, Toronto, ON, Canada M5B 1T8.; University of Toronto, Faculty of Medicine, Department of Psychiatry, Toronto, Canada; St. Michael’s Hospital, Li Ka Shing Knowledge Institute, Keenan Research Centre for Biomedical Research, Toronto, ON, Canada

**Keywords:** memory, hyper memory, savantism, synaesthesia, autistic spectrum disorder, Solomon Shereshevsky, Funes the memorious, memória, hipermemória, savantismo, sinestesia, transtorno do espectro autista, Solomon Shereshevsky, Funes o memorioso

## Abstract

In this paper, we investigated two subjects with superior memory, or hyper memory: Solomon Shereshevsky, who was followed clinically for years by A. R. Luria, and Funes the Memorious, a fictional character created by J. L. Borges. The subjects possessed hyper memory, synaesthesia and symptoms of what we now call autistic spectrum disorder (ASD). We will discuss interactions of these characteristics and their possible role in hyper memory. Our study suggests that the hyper memory in our synaesthetes may have been due to their ASD-savant syndrome characteristics. However, this talent was markedly diminished by their severe deficit in categorization, abstraction and metaphorical functions. As investigated by previous studies, we suggest that there is altered connectivity between the medial temporal lobe and its connections to the prefrontal cingulate and amygdala, either due to lack of specific neurons or to a more general neuronal dysfunction.

## Resumo

Neste artigo, investigamos dois sujeitos com memória superior ou hipermemória: Solomon Shereshevsky, que foi seguido clinicamente por anos por A. R. Luria, e Funes o memorioso, um personagem fictício criado por J. L. Borges. Os sujeitos possuem hipermemória, sinestesia e sintomas do que hoje chamamos de transtorno do espectro autista (TEA). Vamos discutir interações dessas características e seu possível papel na hipermemória. Nosso estudo sugere que a hipermemória em nossos sujeitos sinestésicos pode ser devido às suas características de síndrome do TEA-*savant*. No entanto, esse talento foi acentuadamente diminuído pelo profundo déficit de categorização, abstração e funções metafóricas. Conforme investigado por estudos anteriores, sugerimos que há conectividade alterada entre o lobo temporal medial e suas conexões com o cingulado pré-frontal e amígdala, devido à falta de neurônios específicos ou a uma disfunção neuronal mais geral.

*“If we could recall everything, we would be as incapacitated as if we could not recall at all; a condition to remember is that we must forget.” - William James* 1

In clinical and research studies of the different types of memory, subjects with normal memory function or with memory deficits are usually investigated. The study of persons with exceptional memory abilities that are not reliant on mnemonic techniques or other memory aids has been less explored, with few exceptions,^2^^-^ 4 in spite of the fact that legends about individuals with exceptional memory have been known for centuries.^5^ We will discuss two subjects with incredible memory abilities: the case study of Solomon Shereshevsky^6^ and fictional literary character Ireneo Funes.^7^ A previous communication^8^ describing the similarities in both subjects attracted our attention to these incredible individuals.

In addition to hyper memory, these two subjects also experienced synaesthesia, defined as an event in which stimulation of one sensory modality elicits an involuntary sensation in another sensory modality.^9^ Synaesthesia can be developmental or acquired secondary to a brain insult such as stroke.^10^ Literature suggests that synaesthesia can influence and enhance memory performance,^11^^,^ 12 and that synaesthetes are very creative^13^ and excel in metaphorical functions.^14^ The cases of Luria and Borges are curious because, while both experienced synaesthesia symptoms, they also have similarities in their deficit in abstraction, categorization, understanding, expressing metaphorical thinking and inability to generalize, which are uncommon traits for synaesthetes.

Our aim is to discuss whether Shereshevsky and Funes, besides being synaesthetic, experienced autistic spectrum disorder (ASD) symptoms of the savant type. This idea is supported by a recent review^15^ which suggests that individuals who present with synaesthesia and savants should be investigated for undiagnosed autistic spectrum disorder. Additionally, we will discuss whether they experience dysfunctions of vast cerebral areas such as the temporo-parietal junction, amygdala, insula, and cingulate,^16^ which could explain their hyper memory and their inability to think abstractly, categorize, or think metaphorically.

## Subjects

### Solomon Shereshevsky

This remarkable man with superior memory was followed by the eminent Soviet neuropsychologist A. R. Luria for more than 30 years and whose story was first published in a brief note in the *New York Times* and subsequently as a novel titled “The Mind of a Mnemonist.”^6^

He was able to recall autobiographical information since one year of age. As an adult in Luria’s laboratory, he would recall a long list of words and a lengthy mathematic equation with the same accuracy 12 and 16 years later. The way in which he described his excellent memory was as using a kind of grouping of the words, like the “distribution of houses in a street.”

He describes his remarkable hyper memory and all of his recollections with a variety of multimodal synaesthesia. In his case, noise was creating colours, touch, taste, and smell. Sometimes sounds would appear as visual, tactile, or olfactory synaesthesia. He experienced a confluence of sensory impressions such as, “if I read when I eat, I have a hard time understanding what I am reading, the taste of food floods out other senses.”

Interestingly, Shereshevsky had important deficits in executive functions. He could not understand poetry or metaphors and he was unable to understand abstractions. In his own recollection he states, “I can only understand what I can visualize” and, “In order for me to grasp the meaning of something, I have to see it.” His memory for faces was also impaired. He saw faces as “changing patterns of light and shade” and commonly became confused.

### Ireneo Funes, The Memorious

Ireneo Funes is a fictional character and a Uruguayan gaucho, created by the Argentinian writer J. L. Borges and published in 1942 by a Buenos Aires newspaper La Nación and later in a book named “Fictions.”^7^

This young man suffered a severe traumatic brain injury at the age of 19. He found that the accident had rendered him able to perceive everything in full detail. He was able to remember and have full recollection of the shapes of the clouds on a certain date. He kept these images in his visual memory in the form of a persistent image. He was able to reconstruct a full day’s worth of past memory and spend a full day for the recollection of the memory in an automatic and constant way. As a result of these flooding of memories, it was very difficult for him to sleep. He stated, “To sleep is to be abstracted from the world.”

Funes also experienced synaesthesia, and his hyper memory was associated with somatic perceptions that are described as being all over his body and in his words as “muscular sensations, thermal sensations.”

Borges expressed that previously, since he was young, before the traumatic brain injury, he had characteristics of ASD like a “chronometer”, had exceptional memory for proper names and that he was poor at social interaction. After the accident, it was impossible to eliminate irrelevant memory. He described, “I have more memories in myself alone than all men have had since the world was a world” and, “my memory … is like a garbage dump.” Funes was incapable of platonic ideas or generalities or abstraction, but he was talented in the acquisition and registration of information. For example, he learned to speak English, French, Portuguese and Latin without effort. As his creator Borges suggested, “I suspect, nevertheless, that he was not very capable of thought. To think is to forget differences, to generalize, to abstract. In the overly replete world of Funes there was nothing but details, almost contiguous details.”

## Discussion

Shereshevsky and Funes both experienced synaesthesia associated with hyper memory. We hypothesize that what we now call ASD has contributed to these individual’s superior memory abilities along with their synaesthesia. We suggest this after it was proposed that individuals with hyper memory and synaesthesia should be tested for undiagnosed autism^16^ and because this hypothesis would provide an explanation for the complex symptoms that both these individuals, the real patient Shereshevsky and Funes, the fictional literary character, experienced that are contrary to the developmental synaesthetic experience. For example, both demonstrated severe deficits in categorization, abstraction, and metaphorical thinking.

First, we will explore the possibility that Shereshevsky and Funes had ASD. Both experienced a deficit in executive functions, categorization and generalization, much like individuals with ASD.^17^ They both had trouble creating representations in their mind. Instead, information was represented in a specific and detailed fashion for each individual stimulus, or hyper-specific representation.^18^ A hallmark of ASD also includes impairment in social interaction, communication, stereotyped behaviour, and restricted interests.^19^ Shereshevsky had problems recognizing faces while Funes was known for his social deficits.^7^ Individuals with ASD may also possess memory abilities that differ from neurotypical developing individuals. Various studies suggest that the different degrees of memory performances in ASD depend on the item being processed.^20^ Normal or hyper memory is seen particularly when ASD persons process item-specific information,^21^ while poor memory is observed when they perform tasks requiring mental organizations such as semantic clustering or other subjective approaches.

While both ASD and synaesthesia are typically conditions involving functional connectivity, ASD is likely characterized by local over-connectivity and long range under-connectivity,^22^ while developmental synaesthesias are characterized by hyper connectivity in different sensory systems.^16^^,^ 23 ^,^ 24 Both conditions occur developmentally and reportedly share similar genes.^25^ These individuals tend to have hyper memory for synaesthesia-inducing materials, but only for these materials. It is suggested that this may be due to ASD and synaesthesia being comorbidities, since a common symptom of ASD includes obsessive tendencies toward one particular topic.^16^

Single case studies of patients with medial temporal lobe (MTL) lesions involving both hippocampi were the origin of the discovery of various memory systems in humans.^26^^-^ 28 The current knowledge of memory consolidation and retrieval suggests that the interaction of the entorhinal and hippocampal areas in the MTL are essential in the acquisition of memory; the entorhinal cortex may contribute to the sensory integration and mnemonic processes, providing the hippocampus with cortical sensory inputs and is thought to play roles in sensory integration, memory formation and spatial navigation. The entorhinal cortex acts as a “gatekeeper” of cortical memory networks, particularly to the prefrontal-cingulate cortex.^29^^,^ 30 It is suggested that the encoding of declarative memory is done in an abstract form; the single memory of animals, objects, faces and places creates an abstract encoding, or a prototype, that is used to store memory. As a result, we remember concepts and not irrelevant details.^31^ It may be possible that Shereshevsky and Funes may recall this bulk of memory, but with no discrimination or the natural human filter of categorization.

As discussed above, ASD has been associated with dysfunctions among many neural systems including brain areas such as the MTL, parietal lobe, amygdala and cingulate cortex^23^ which is hypothesized to be due to either a lack of a specific type of neuron^32^ or to a more general dysfunction such as the mirror neuron system.^33^^-^ 35 These neuronal dysfunctions can lead to deficits in categorization, abstraction and metaphorical functions, as seen in our subjects. Additionally, this dysfunction can lead to ASD-like symptoms such as a tendency to hyper systematize^36^, process information locally instead of globally^37^ and an inability to create a prototype for remembering information.^28^ More research should be done to draw conclusions about the involvement of MTL and ASDs in hyper memory in these remarkable individuals.^38^
